# Recent Advances in the Synthesis of 3,4-Dihydropyran-2-Ones Organocatalyzed by *N*-Heterocyclic Carbenes

**DOI:** 10.3390/molecules28093743

**Published:** 2023-04-26

**Authors:** Camilo Morales-Manrique, Edwin A. Baquero, James Guevara-Pulido

**Affiliations:** 1 Estado Sólido y Catálisis Ambiental (ESCA), Departamento de Química, Facultad de Ciencias, Universidad Nacional de Colombia, Carrera 30 No. 45-03, Bogotá 111321, Colombia; 2 INQA, Química Farmacéutica, Facultad de Ciencias, Universidad El Bosque, Bogotá 11001, Colombia

**Keywords:** 3,4-dihydropyran-2-ones, *N*-heterocyclic carbenes, cycloaddition, organocatalysis

## Abstract

In recent years, *N*-heterocyclic carbenes (NHC) have gained recognition as versatile molecules capable of acting as organocatalysts in various reactions, particularly through the activation of aldehydes via Breslow-type adducts. This organocatalytic activation has enabled the production of numerous 3,4-dihydropyran-2-ones and related derivatives. In this review, we provide an overview of the production of 3,4-dihydropyran-2-ones and derivatives via organocatalytic processes involving NHCs over the past eight years. These processes involve the use of a diverse range of substrates, catalysts, and reaction conditions, which can be classified into [4+2]-and [3+3]-type cycloadditions, primarily aimed at synthesizing this skeleton due to its biological activity and multiple stereocenters. These processes are scaled up to the gram scale, and the resulting products are often directed towards epimerization and functionalization to produce more complex molecules with potential applications in the biological field. Finally, we provide a perspective and the future directions of this topic in organic synthesis.

## 1. Introduction

**Figure 1:**
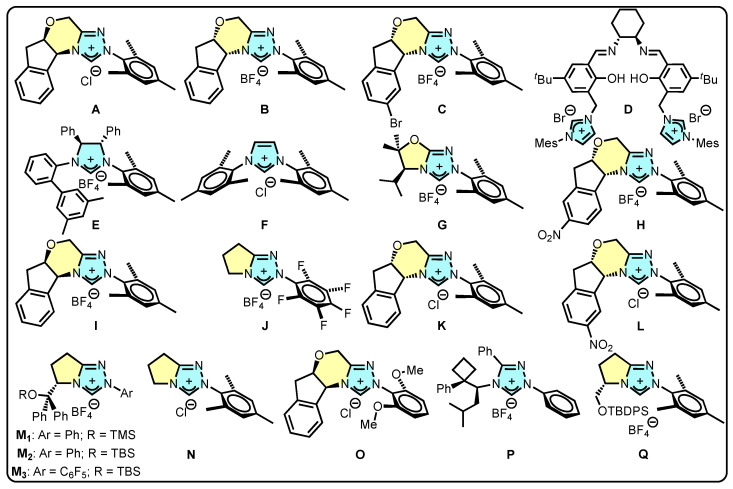
Imidazolium and triazolium salts employed as precatalysts in the present review.

Also known as enol δ-lactones, 3,4-Dihydropyran-2-ones have become increasingly popular due to their biological activity [1], presence in various pharmaceutical products [2], and usefulness in organic synthesis. These compounds provide a versatile platform for accessing functionalized enones, γ-lactones, cyclic enamines, and 2-pyrones, among others [3], making them a subject of significant interest in natural product extraction and novel synthesis. One of the most commonly used methods for producing 3,4-dihydropyran-2-ones is through organocatalysis with *N*-heterocyclic carbenes (NHCs) [4,5,6,7,8,9,10,11,12,13,14]. Research in this field has been increasing since the publication of the annulation of tropones and enals via homoenolate in 2006 [15]. Although this catalytic process has been reviewed extensively [7,9,16,17,18,19,20,21,22], including a recent review by Albanese and Gaggero in 2014 [23], research in this field continues to advance, and this review aims to highlight recent developments in the organocatalytic production of 3,4-dihydropyran-2-ones using NHCs (Figure 1).

**Scheme 1:**
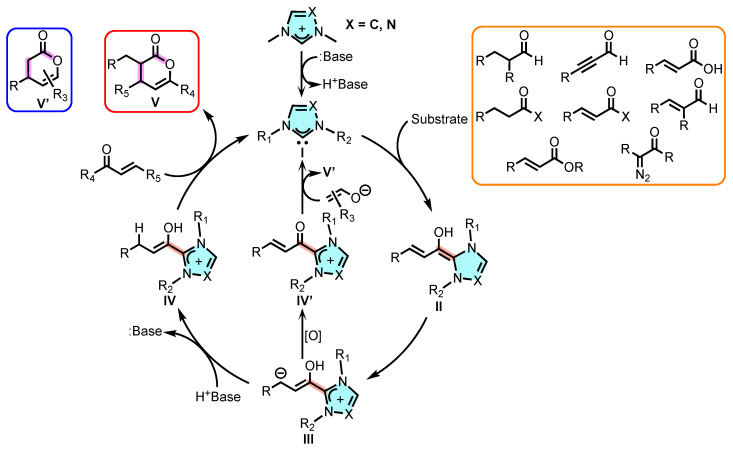
Catalysis with NHC from different reagents for the production of 3,4-dihydropyran-2-ones.

NHCs are easily produced in situ from the deprotonation of the corresponding azole (imidazolium and/or triazolium salts). Their versatility in modulating stereoelectronic properties through the modification of the backbone and *N*-substituents has generated considerable interest their role as catalysts in various types of reactions [9,10], either alone or coordinated to metals [24,25,26,27,28]. In the context of 3,4-dihydropyran-2-one synthesis, NHC catalysis involves [4+2]- and [3+3]-type cycloadditions, which, depending on the established reaction mechanisms, rely first on the in situ formation of the free NHC (**I**) with a subsequent nucleophilic attack to the corresponding substrate to yield the Breslow intermediate (**II**). Further, a rearrangement allows the formation of a homoenolate species **III** that, depending on the reaction conditions, is able to form either the azolium enolate species **IV** or the α, β-unsaturated acylazolium species **IV′** (when oxidant conditions are used) as key reactive intermediates. Finally, these species are converted to the corresponding dihydropyranones **V** and **V′**, respectively (Scheme 1). The key reactive intermediates species can be generated from a variety of functional groups, including aldehydes, ketones, carboxylic acids, and acyl halides, allowing the diverse production of the skeleton to meet different synthetic needs and challenges.

In this review, we provide an overview of recent advances in the NHC-organocatalyzed synthesis of dihydropyranones from a variety of substrates. To facilitate practical applications, we have organized our discussion by the chemical functions involved in the reaction. Specifically, we will begin by examining recent reports on the use of α, β-unsaturated aldehydes as substrates, followed by a review of reactions involving saturated aldehydes. We will then cover reactions using ynals, ketones, and finally carboxylic acids and derivatives as substrates. By presenting this information in a clear and organized manner, we hope to provide readers with a comprehensive understanding of the latest developments in this field, as well as insights into future directions for research in the synthesis of dihydropyranones.

## 2. α, β-Unsaturated Aldehydes as Reagents for Dihydropyranones

**Scheme 2:**
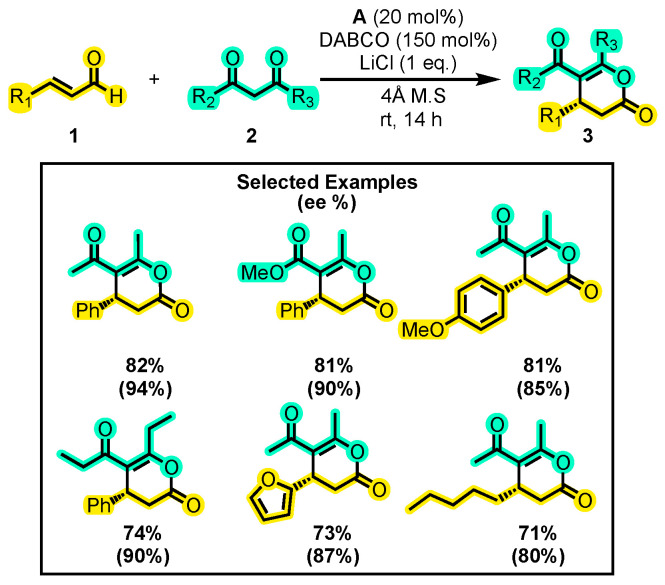
Synthesis of trisubstituted dihydropyranones from α, β-unsaturated aldehydes and 1,3-dicarbonyl compounds. Adapted from reference [32].

Polyfunctional substrates that feature a double bond conjugated with an aldehyde group, also known as α, β-unsaturated aldehydes, are considered valuable synthetic building blocks. Their high reactivity makes them versatile tools for the targeted synthesis of important natural compounds and other molecular scaffolds [29,30,31]. Regarding their reactivity, these molecules are known for readily yielding the intermediate species required for producing enol δ-lactones. In this sense, many studies have focused on cinnamaldehyde as the main reactant. Thus, Xie and co-workers developed an efficient aerobic method for synthesizing trisubstituted dihydropyranones **3** with high yield and enantiomeric excess [32]. This study utilized the reactivity of α, β-unsaturated aldehydes **1** towards 1,3-dicarbonyl compounds **2** under specific conditions, including the use of DABCO, THF, LiCl, and a 4 Å molecular sieve along with catalyst **A**. While other catalysts, bases, solvents, and sieve sizes were tested, the conditions outlined in Scheme 2 yielded the best results. The authors also explored the substrate scope, finding that both aliphatic and aromatic substituents on the substrates worked well in the reaction. The same core structure, with similar substitutions at positions 4, 5, and 6, have also been synthesized using isothiourea as a catalyst. Nonetheless, the resulting yield and enantiomeric excess were significantly lower [33].

**Scheme 3:**
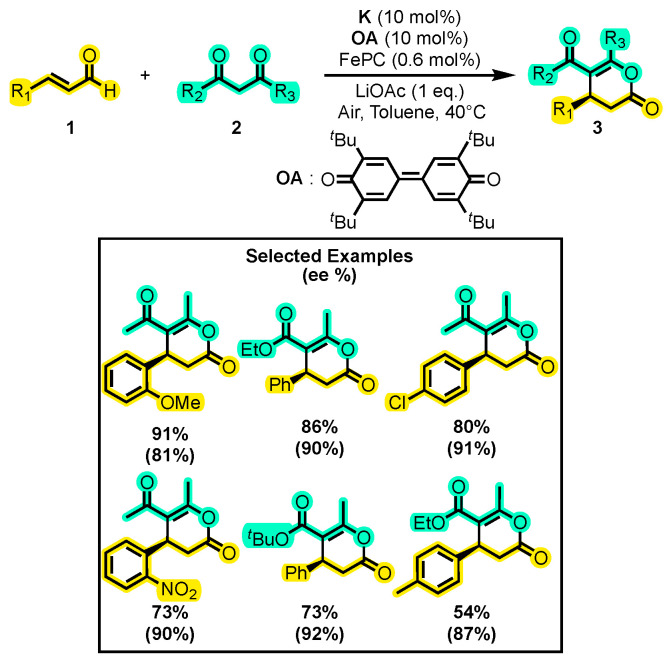
Synthesis of dihydropyranones from α, β-unsaturated aldehydes and 1,3-dicarbonyl compounds with a system of electron transfer mediators. Adapted from reference [34].

Axelsson and co-workers also employed an enal **1** and a 1,3-dicarbonyl compound **2** to perform a [3+3] cyclization (Scheme 3) [34]. However, they implemented an electron transfer mediator (ETM) system that allowed the formation of the unsaturated acylazolium [35] from the homoenolate according to the proposed catalytic cycle. Iron(II) phthalocyanine (FePC) and air passage play a key role in the reaction yield as a pure O_2_ atmosphere does not generate any product. The use of lithium acetate dihydrate as a base in this study is not frequently observed in other research works. The reaction maintained the yield (above 54%) regardless of the type of substitution used. Although the utilization of quinone as an oxidizing agent (**OA**) is frequently employed in many investigations, and dinitrobenzenesulfonic carbamate [36] and polyhalides [37] have also been demonstrated to serve as useful OAs.

**Scheme 4:**
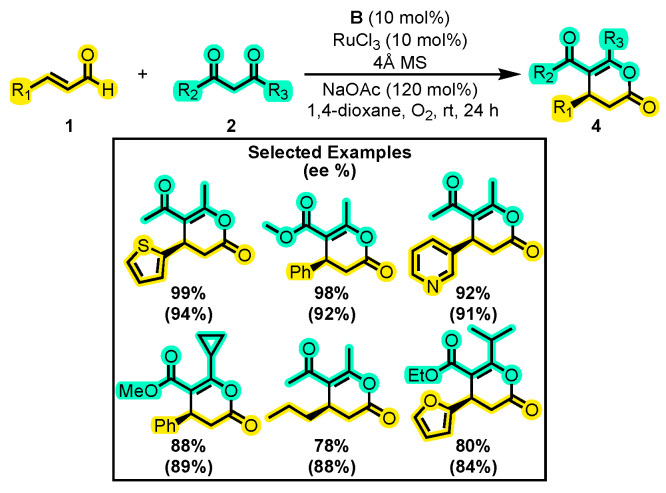
Synthesis of dihydropyranones from α, β-unsaturated aldehydes and 1,3-dicarbonyl compounds through synergistic catalysis. Adapted from reference [38].

Likewise, Wang and co-workers developed a method for the formation of chiral δ-lactones using a synergistic catalysis approach in which NHC and Ru work together [38]. RuCl_3_ was used as the metallic source, as it favors the formation of an α, β-unsaturated species over that of a β-protonated species. The best reaction conditions involved the use of NaOAc as a base, 1,4-dioxane as a solvent, and the catalytic system was formed by the mixture of RuCl_3_ and **B** (Scheme 4). The authors were able to perform the reaction with different substituents at either the aldehyde or the dicarbonyl compound with yields up to 99% and an enantiomeric excess of about 94%. The authors studied a wide range of groups, including aryl, vinyl, heterocycles, and complicated skeletons. The authors also noted that the replacement of the 1,3-dicarbonyl compound with β-ketosters did not prevent annulation, whereas β-keto amides did.

It should be noted that products **3** and **4** have been derivatized and subjected to reactions such as epoxidations [39], alkylations [40], and ring-openings [41] that preserve chirality.

**Scheme 5:**
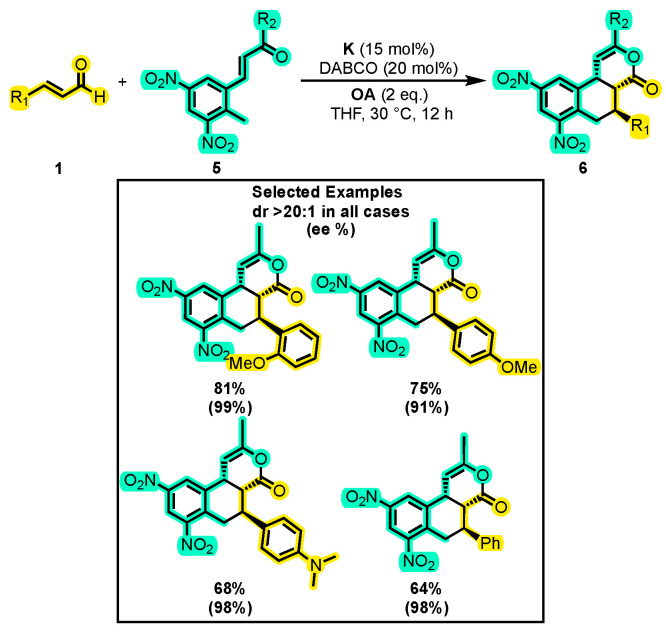
Synthesis of chiral tricyclic δ-lactone from α, β-unsaturated aldehydes and α, β-unsaturated ketones. Adapted from reference [42].

On the other hand, tricyclic δ-lactone **6** is a highly desirable product due to its ability to undergo various functionalizations, including the reduction of the nitro groups and the double bonding of the lactone, as well as ring openings and contractions (Scheme 5). In this sense, Mukherjee and co-workers achieved the synthesis of this skeleton by substituting the 1,3-dicarbonyl compound with an α, β-unsaturated ketone under oxidative conditions [42]. Initially, the reaction conditions involved the use of DBU, a quinone as the OA, and catalyst **A** dissolved in THF. These conditions allowed the authors to obtain an enantioselectivity of 98% and a yield of 51%. However, when assays were performed with different bases (*^t^*BuOK, NEt_3_, DMAP, Cs_2_CO_3_, and DABCO), they found that only DABCO improved the yield to 58%. Changing the solvent did not improve the results. With the optimal reaction conditions, various substitutions were performed on the radicals of enal **1** and dinitrotoluene derivative **5** (Scheme 5). Notably, the absence of the two nitro groups resulted in only trace amounts of compound **6**. Furthermore, when R_2_ was a methyl, ethyl, or cyclopropyl group, the yield was maintained, while yields exceeding 70% were obtained when R_1_ was a 4-OMe-Ph or 2-OMe-Ph group. It should be noted that the diastereoselectivity was consistently higher than 20:1 in all tests carried out.

**Scheme 6:**
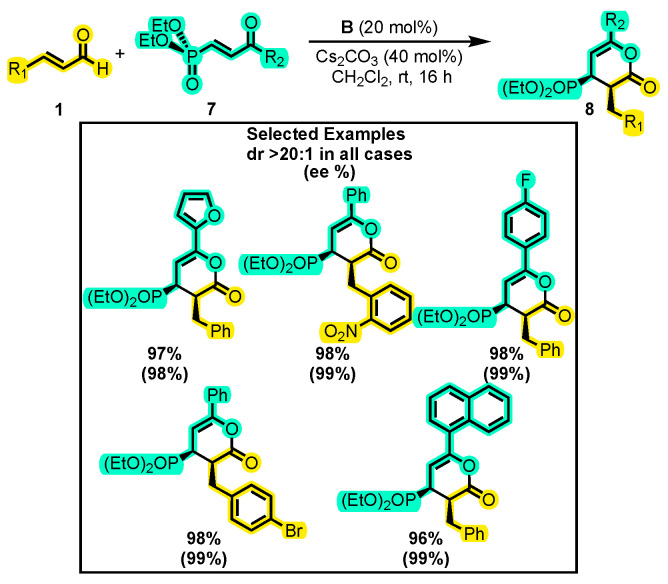
Synthesis of phosphorylated δ-lactones from α, β-unsaturated aldehydes and β-phosphorylenones. Adapted from reference [43].

Verma and co-workers developed a novel approach using an enone as a nucleophile to obtain phosphorylated δ-lactones **8**, which can serve as precursors for amides and ketophosphorylated esters (Scheme 6) [43]. In this study, single enals **1** and β-phosphorylated enone **7** were reacted using catalyst **B**, Cs_2_CO_3_ as a base, and CH_2_Cl_2_ as the solvent. The enals and enones were modified with various aromatic ring substitutions including halogens, MeO, Me, NO_2_, etc. The reaction provided high yields, around 90%, and excellent enantiomeric excess, close to 99%, showing a good tolerance to functional groups. This approach represents a significant advance in the development of phosphorylated δ-lactones, offering potential as building blocks for the synthesis of various biologically active molecules. In fact, a lactone skeleton with the same substitution pattern as **8** has been proposed as a new pharmacophore for dual PPARγ/GR modulators with therapeutic potential against human metabolic diseases [44].

**Scheme 7:**
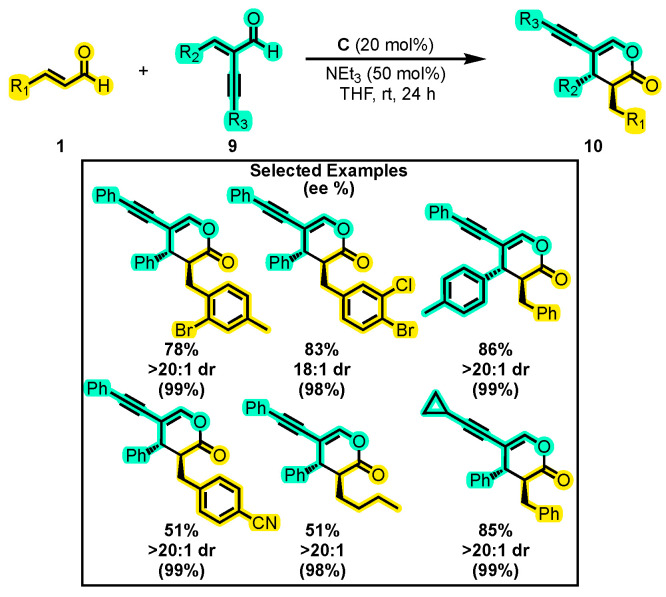
Synthesis of 3,4,5-trisubstituted dihydropyranones from two enals in a [2+4] annulation reaction. Adapted from reference [45].

Peng and co-workers also employed catalyst **B** in their reactions, as shown in Scheme 7. However, they discovered that the substitution of the aromatic ring with Br (to form catalyst **C**) significantly enhanced the yield compared to other catalysts tested during the optimization of the reaction. The chemoselective coupling of two enals was carried out using NEt_3_ as a base and THF as a solvent. The first enal was **1** and the other was α-substituted with an alkyl group **9** to yield a 3,4,5-trisubstituted dihydropyranone **10** (Scheme 7) [45]. The reaction worked well for a wide range of groups with yields ranging from 51–97% and enantiomeric excesses of up to 99%. It is also noteworthy that replacing the α-alkynyl group with other groups such as vinyl, phenyl or cyano groups did not result in cross-reactions [2+4]. Additionally, the presence of the alkynyl group as a substituent in dihydropyranones makes it a versatile molecule that can undergo various addition reactions, resulting in multi-functionalized alkenes.

**Scheme 8:**
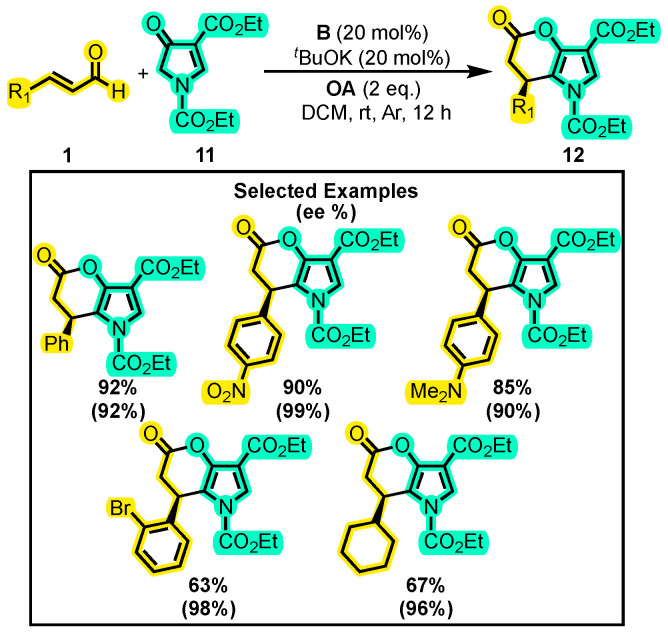
Synthesis of pyrrole-substituted dihydropyranones from α, β-unsaturated aldehydes and pyrrol-4-ones. Adapted from reference [46].

Wu and Yetra carried out a different approach compared to the previous studies by utilizing heterocyclic compounds as nucleophiles in [3+3] annulations. In 2020, they developed a method for the annulation of enal **1** with pyrrole-4-ones **11** (Scheme 8) [46]. The reaction employed *^t^*BuOK as a base, CH_2_Cl_2_ as a solvent, and quinone as the OA with catalyst **B**. The yields of **12** ranged from 50–98% under these conditions, while the ee% was above 90% for more than 25 examples. The authors proposed that the catalytic cycle occurs through the Breslow intermediate, followed subsequently by the α, β-unsaturated acylazolium intermediate. At this point, a 1,4 addition or a 1,2 addition can occur, ultimately leading to an intramolecular acylation to obtain a dihydropyranone derivative.

**Scheme 9:**
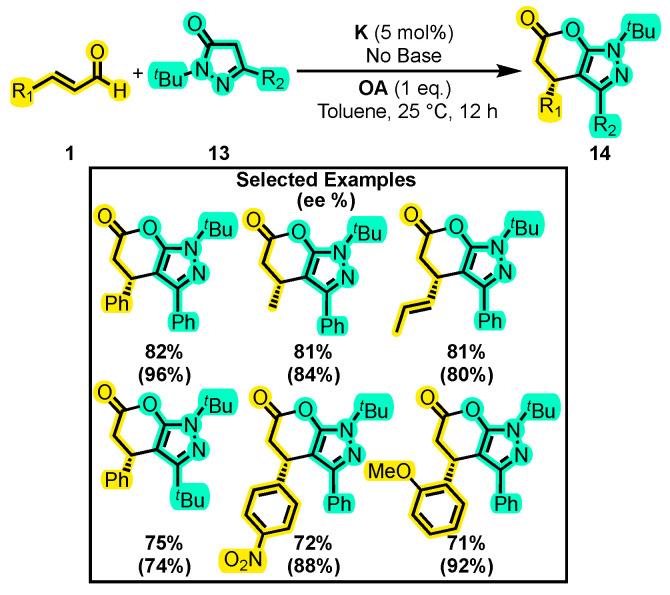
Synthesis of dihydropyranones from α, β-unsaturated aldehydes and pyrazolones. Adapted from reference [47].

In another report, Yetra and co-workers carried out an enantioselective annulation reaction of α, β-unsaturated aldehydes **1** with pyrazolones **13** (Scheme 9) [47]. Pyrazolone derivatives have been extensively studied for their antimicrobial, antitumor, and anti-inflammatory properties [48,49]. These compounds have demonstrated potential in various fields, such as in the identification of agonists of G protein receptor 39 through homology modeling [50], showcasing their versatility and research potential. To determine the optimal reaction conditions, a set of solvents and bases were tested. Interestingly, it was found that the reaction in the absence of a base achieved a yield of 55% of **14**, which is comparable to that achieved with bases such as Na_2_CO_3_ and DABCO. Therefore, the scoping of the substituents was carried out without the use of a base. Yields of up to 82% and an enantiomeric excess of 96% was obtained with different substitutions. Although the substitution of pyrazolone with oxazolone and α-lactone was considered, the reaction did not yield satisfactory results. Additionally, structures similar to that of **14** have demonstrated inhibitory activity against phosphoinositide-dependent protein kinase 1 (PDK1) [51]. Therefore, the large number of molecules obtained in this research opens the door for the exploration of this scaffold and its potential biological activities.

**Scheme 10:**
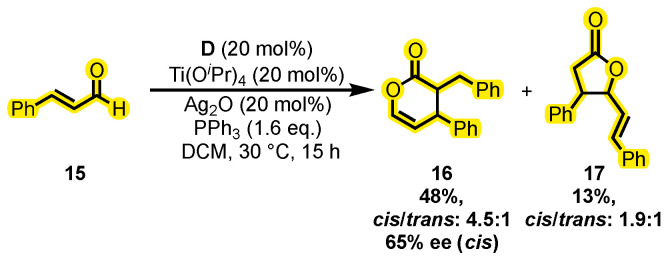
Synthesis of dihydropyranone **16** from self-condensation of cinnamaldehyde. Adapted from reference [52].

An investigation using novel procedures was conducted by Latendorf and co-workers, in which an Ag–NHC complex catalyzed the formation of dihydropyranones **16** from cinnamaldehyde **15** (Scheme 10) [52]. In this process, the addition of PPh_3_ was essential to obtain the desired product and to prevent the formation of the competing γ-lactone **17**. Although the dihydropyranone was obtained with a maximum yield of 48% and 66% ee in its major diastereoisomer, this report provides a potential pathway for obtaining chiral dihydropyranones using a single substrate through the combined action of NHC and metals. Product **16** has been used as a substratein the synthesis of yohimbine-type alkaloids, a family of pentacyclic indole compounds with a broad pharmacological spectrum [53].

**Scheme 11:**
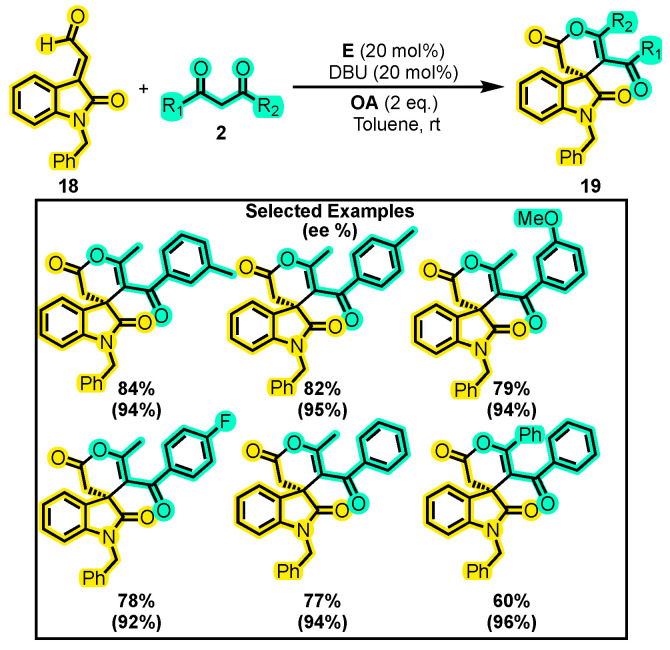
Synthesis of spirooxindole δ-lactones from isatin derivatives and 1,3-dicarbonyl compounds. Adapted from reference [54].

On the other hand, a method published by Lin and co-workers represented a breakthrough from previous studies where the α, β-unsaturated aldehyde was typically β-substituted with small electron-donating and electron-withdrawing groups. Specifically, the authors developed a highly efficient protocol for synthesizing spirooxindole δ-lactones **19** from isatin-derived enals **18** and 1,3-dicarbonyl compounds **2** (Scheme 11) [54]. The authors found that the optimal conditions consisted of using DBU as a base, toluene as a solvent, catalyst **E**, and a reaction time of just one hour (one of the shortest reported). When substituents on the dicarbonyl compounds were methyl and substituted phenyl moieties, the yields of the products reported ranged from 77–84% with enantiomeric excesses of up to 96%. While the substitution of the benzyl group in **18** with methyl, allyl, and *n*-butyl moieties was also tested, poor yields were obtained.

**Scheme 12:**
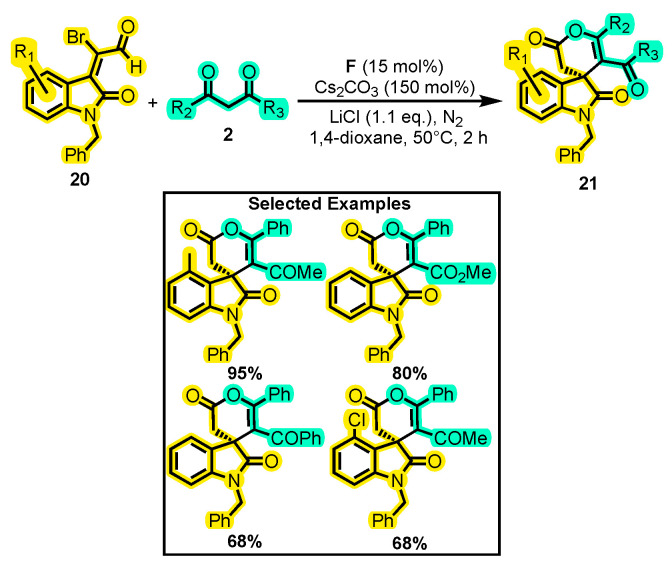
Synthesis of dihydropyranones from α-substituted isatin derivates and 1,3-dicarbonyl compounds. Adapted from reference [55].

Xu and co-workers conducted a similar study to the one mentioned above, where they investigated the reactivity of α-substituted isatin derivative **20** towards 1,3-dicarbonyl compounds **2** with the aim to obtain spirooxindole δ-lactones **31** (Scheme 12) [55]. Initially, they screened different catalysts and found that only catalyst **F** (in the presence of THF and DBU) gave a yield of over 10%, hence the other catalysts were eliminated. The authors then explored different solvents and bases and determined that Cs_2_CO_3_ and 1,4-dioxane resulted in a 42% yield. By adding a Lewis acid (LiCl) to the reaction, the yield was further increased to 63%. Finally, the researchers studied the substrate scope of the reaction by using different substitutions on the substrates under the optimized conditions, achieving yields up to 95%, except when cyclohexane-1,3-dione was used, which did not produce any reaction. Further, they carried out a preliminary study of the enantioselectivity of the reaction by using chiral catalyst **A**. However, the desired product was obtained in a moderate yield (42%) with no enantioselectivity (0% ee).

**Scheme 13:**
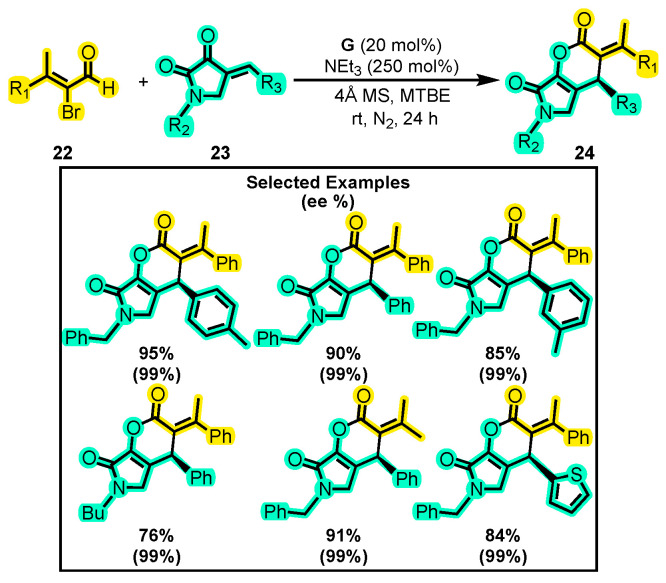
Synthesis of dihydropyranones from α-substituted enals with dioxopyrrolidines. Adapted from reference [56].

In contrast to Xu’s approach, Liu and co-workers developed novel [4+2] annulations from α-substituted enals. They reported the synthesis of α-alkylidene-δ-lactone **24** from α-bromoenals **22** and dioxopyrrolidines **23** (Scheme 13) [56]. The reaction conditions were optimized by testing various catalysts, bases, and solvents, among which catalyst **G**, trimethylamine, and tert-butyl methyl ether (MTBE) provided the best yields. The substitution variations on dioxopyrrolidines retained a high enantiomeric excess of 99%, but the yield ranged from 95–88%, with the -Ph, -PhMe, and -Ph(MeO)_2_ groups being the most effective. The substitutions on α-bromoenals also maintained high enantiomeric excess (99%) but with a significant decrease in yield (91–67%). Similar research was conducted by Shen and co-workers, who achieved a yield of 82% and an excellent %ee (>99%), even on a gram scale [57]. Molecules such as **24** and derivatives are highly important in synthetic chemistry as they provide fast and simple access to the synthesis of alkaloids with great therapeutic potential [58].

**Scheme 14:**
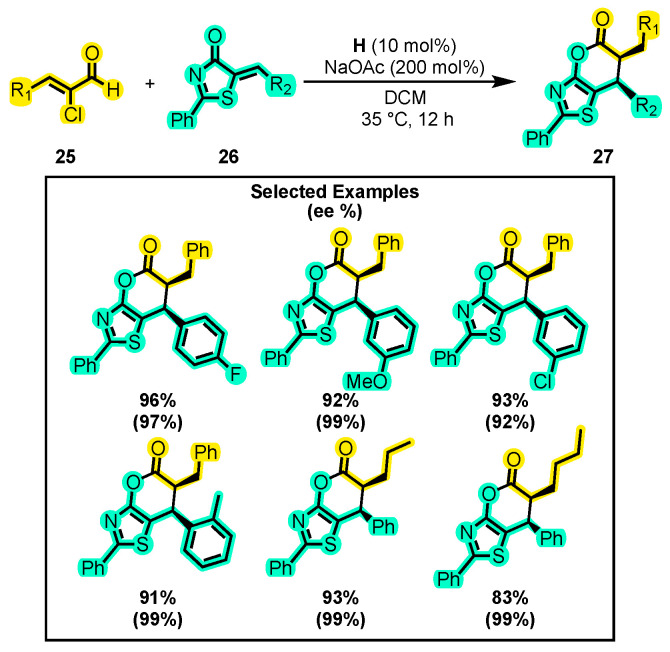
Synthesis of bicyclic dihydropyranothiazoles from α-substituted enals and alkenylthiazolones. Adapted from reference [59].

In a related study, the reactivity of α, β-substituted aldehyde **25** with 5-alkylthiazolones **26** was investigated by other researchers, using imidazolium salt **H** as a catalyst (Scheme 14) [59]. The reaction resulted in the formation of bicyclic dihydropyranothiazoles **27** with excellent enantiomeric excesses and high yields. Although the study did not evaluate the effects of solvents or the catalyst, CH_2_Cl_2_ and **H** were used in all catalytic tests. However, the effect of the base was studied, and significant yields were obtained using DMAP (97%), DIPEA (97%), DABCO (92%), K_2_CO_3_ (98%), and NaOAc (94%). Notably, NaOAc had slightly a lower yield than K_2_CO_3_, but it produced a higher diastereomeric ratio, exceeding 20:1. Additionally, the authors studied the substrate scope by varying the substitution of 5-alkenylthiazolone **26** with substituted aromatic rings. Specifically, when the substituent on the aromatic ring was -4-F, -3-OMe, -3-Cl, or -2-Me, they found 96%, 92%, 93%, and 91% yields, respectively. In addition, when the groups on the α-chloroaldehyde **25** were propyl or butyl groups, the yields obtained were 93 and 83%, respectively, presenting an enantiomeric excess of 99%. The importance of developing skeletons such as that of **27** lies in diversifying the thiazole group, which is well known to be present in several pesticides [60] and to have a wide range of pharmacological activities [61,62].

**Scheme 15:**
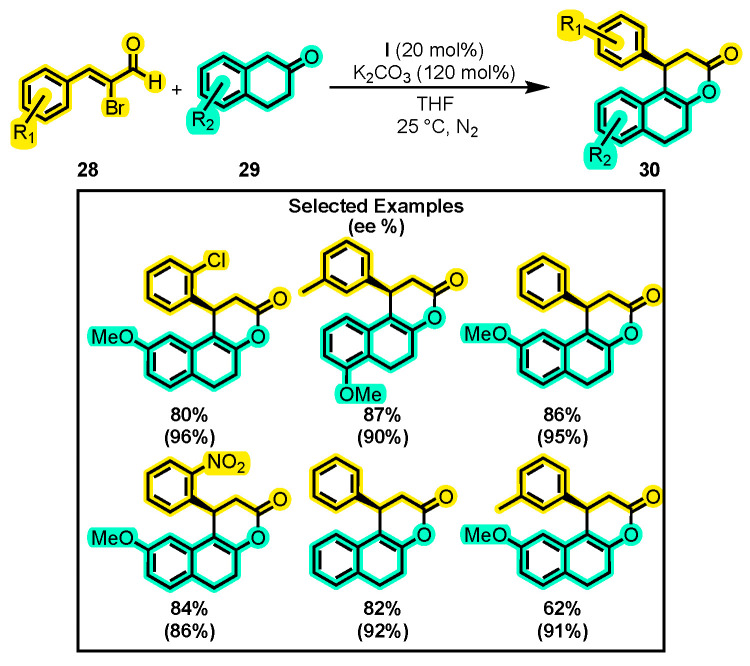
Synthesis of tricyclic dihydropyranones from α-substituted enals and β-tetralones. Adapted from reference [63].

Li and co-workers developed a novel method to synthesize tricyclic dihydropyranones **30** by using α-bromoenal **28** and β-tetralones **29** via a [3+3] annulation (Scheme 15) [63]. The study involved a comprehensive investigation of various reaction conditions, including temperature (−5 °C to 45 °C), bases (NEt_3_, DABCO, K_2_CO_3_, NaOAC, and K_2_CO_3_), solvents (THF, toluene, CH_2_Cl_2_, DME, and 1,4-dioxane), and catalysts. The authors also evaluated the tolerance of the reaction towards substitutions on the aromatic rings of the substrates. The obtained yields ranged from 62% to 90%, while the enantiomeric excess was between 81% to 96%. Interestingly, the reaction yielded undesired products when α-tetralone was used instead of β-tetralone. However, β-indanone reacted favorably in the presence of DABCO and toluene. Previously, products such as **30** in their hemiacetal form were obtained using the Hayashi–Jørgensen catalyst [64], with yields and enantioselectivities comparable to those found by Li and co-workers.

**Scheme 16:**
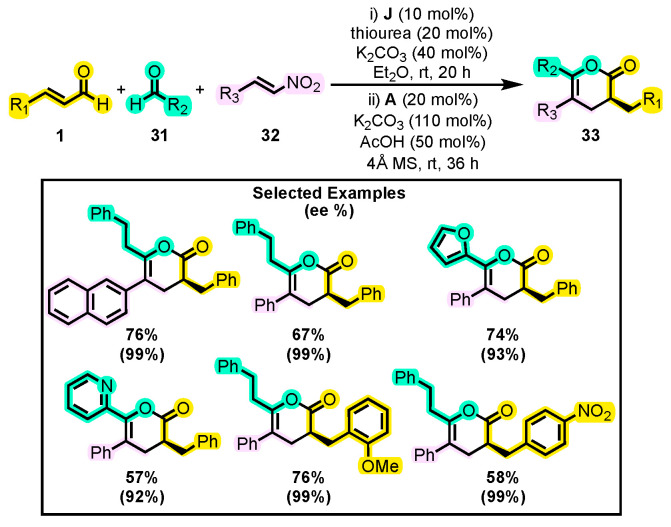
Synthesis of 5,6-trisubstituted dihydropyranones from aldehyde, enal and nitroalkene derivatives. Adapted from reference [65].

Until now, we have mainly discussed reactions involving two substrates, where one of them has been always an α, β-unsaturated aldehyde. Due to their electrophilic and nucleophilic characteristics, they can participate in [4+2] or [3+3] annulations with NHCs as a catalyst. However, Fuchs and co-workers introduced an innovative method for synthesizing 3,5,6-trisubstituted dihydropyranones **33**, involving the use of three substrates and two NHCs as a catalytic system [65]. Initially, they performed the reaction using two aldehydes **31** (one of them being unsaturated **1**), a β-nitroalkene **32**, and catalysts **J** and **A** (Scheme 16). The presence of thiourea (a nitroalkene activator), diethyl ether (solvent), and a second catalyst was found to be indispensable to maintaining the yield of the reaction (72%). Furthermore, it was shown that the addition of a chiral catalyst produced a product with good enantiomeric excess. The substrate scope was then evaluated, and a wide range of substituents was tested. Some substituents such as naphthyl, furanyl, and butyl enhanced the initial yield, which was superior to that reported in other studies that attempted to synthesize this core through an alternative pathway [66]. Finally, a hydrogenation of the double bond was performed on a product with two phenyl substituents and a pyridyl group, yielding an “*all-cis*” δ-lactone to demonstrate the suitability of this synthetic methodology. A significant advantage of compounds with a structure similar to that of **33** is their low tendency to decompose due to moisture and their high stability in column chromatography systems on silica gel [67], making them potentially useful in the field of chromatography as chiral modifiers. Additionally, dihydropyranones with the substitution pattern of that of **33** have been extracted from plants distributed in Asia that are attributed with medicinal benefits [68,69].

**Scheme 17:**
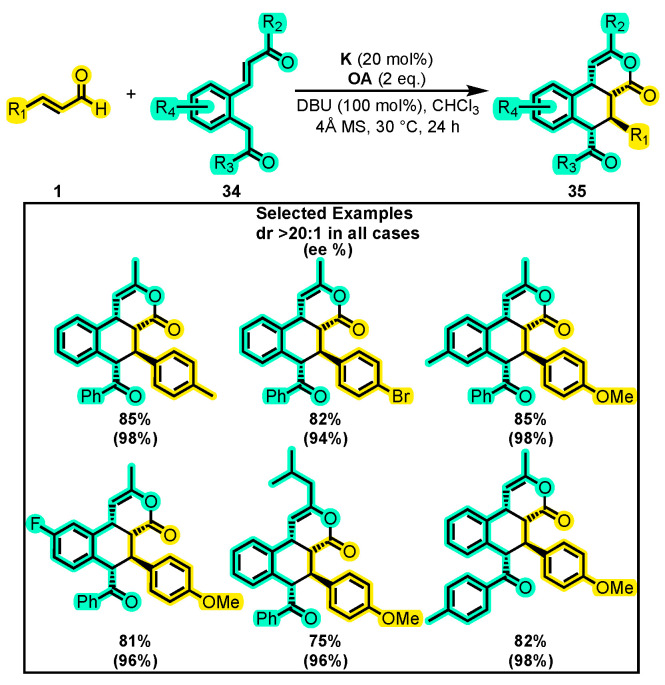
Synthesis of tetra-substituted tetralines **35** from α, β-unsaturated aldehydes and enones. Adapted from reference [74].

On the other hand, Biju and co-workers recently reported a new method for the synthesis of biologically relevant [70,71,72,73] tetra-substituted tetralines **35**, which contain four contiguous stereocenters. This approach uses an NHC-organocatalyzed azolium-enolate cascade reaction from the corresponding enals **1** and enone **34** derivatives with **K** as a catalyst under oxidative conditions (Scheme 17), resulting in a highly stereoselective and broad-scope synthesis [74]. After optimizing the reaction conditions, the authors investigated the scope of the reaction by using different enals and enone derivatives. They discovered that various enals with electronically different groups at the *ortho*, *meta* or *para* positions of the β-aryl ring furnished the tetraline derivatives with high levels of selectivity (>20:1 dr and >86% ee in all cases) and isolated yields. Similarly, the authors found that different substituents on the enone moiety were well tolerated under the optimized reaction conditions, providing the target tetralines without compromising the yield and selectivity. Notably, they observed a negative non-linear effect [45,75] on the change in ee values of one of the products with the change in ee values of the catalyst **K**. The authors attributed this observation to the possibility of more than one catalyst being involved in the enantio-determining step of the reaction, as well as the possible Brønsted base activation of the enone using the NHC derived from **K** under the reaction conditions for the facile Michael addition to catalytically generated α, β-unsaturated acylazoliums.

**Scheme 18:**
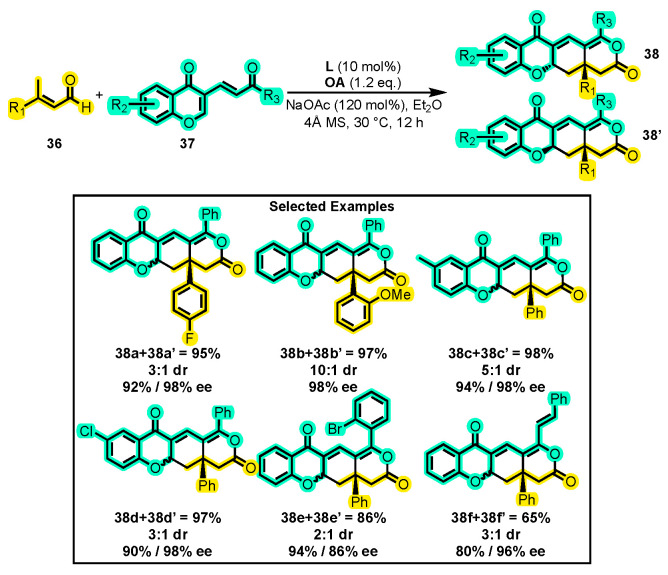
Synthesis of multicyclic dihydropyranones from enals and dienones. Adapted from reference [76].

Further, Chi and co-workers disclosed a new cascade reaction that utilizes NHC organocatalysis to facilitate the synthesis of complex multicyclic lactones that are otherwise difficult to prepare. The reaction involves the use of β-methyl enals **36** and dienones **37** through the first NHC-catalyzed 1,6 addition of acylazolium vinyl enolate γ-carbons. The reaction enables the construction of structurally complex molecules containing chiral quaternary carbon centers, which are typically challenging to prepare. The reaction proceeds by forming complex ring-fused dihydropyranones **38** and **38′** in excellent yields and selectivities using catalyst **L** and NaOAc as a base at room temperature for 12 h (Scheme 18) [76]. The authors also conducted a study of the scope of the process and found that the reaction was tolerant to various substitutions on the corresponding enal **36** bearing aryl rings, including both electron-donating and -withdrawing moieties, as well as heteroaryl and aliphatic substituents. Moreover, the reaction proceeded well with different substitution patterns on the corresponding dienone substrates without a detriment to the yield or selectivity, regardless of the electronic properties of the substituents. The authors were also able to scale up the synthesis of one of the products on a 1 mmol scale. To demonstrate the generality of the reaction, the authors were able to obtain fused-ring dihydropyranones from dienones bearing substituted pyranone moieties.

**Scheme 19:**
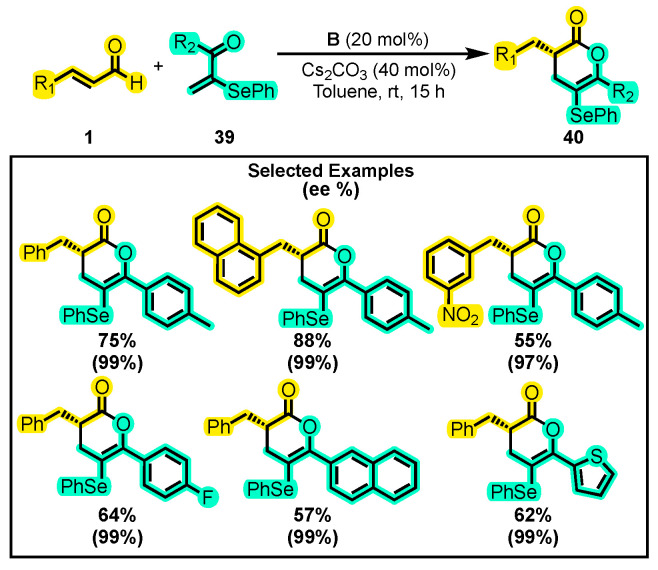
Synthesis of chiral selenylated dihydropyranones **40** from α, β-unsaturated aldehydes and selenyl vinyl ketones. Adapted from reference [80].

Finally, in 2022 Tiwari and co-workers successfully developed the first enantioselective method for synthesizing selenylated dihydropyranones. This type of molecule is of particular interest due to the well-known augmentation of physical, chemical, and biological properties through the functionalization of organic molecules with selenyl groups [77,78,79]. By using NHC organocatalysis, the authors achieved high yields and excellent enantioselectivities of chiral selenylated dihydropyranones **40** from α, β-unsaturated aldehydes **1** and selenyl vinyl ketones **39** (Scheme 19) [80]. The optimal reaction conditions were determined to be Cs_2_CO_3_ as a base and **B** as a catalyst in toluene at room temperature for 15 h. The authors briefly outlined the scope of the reaction, demonstrating that their protocol tolerated various functional groups on both **1** and **39** substrates without compromising the yield and enantioselectivity of the products. Notably, electron donating or withdrawing groups on both substrates were successfully converted to the corresponding dihydropyranones in good to excellent yields and enantiomeric excesses.

## 3. Saturated Aldehydes as Reagents for Dihydropyranones

**Scheme 20:**
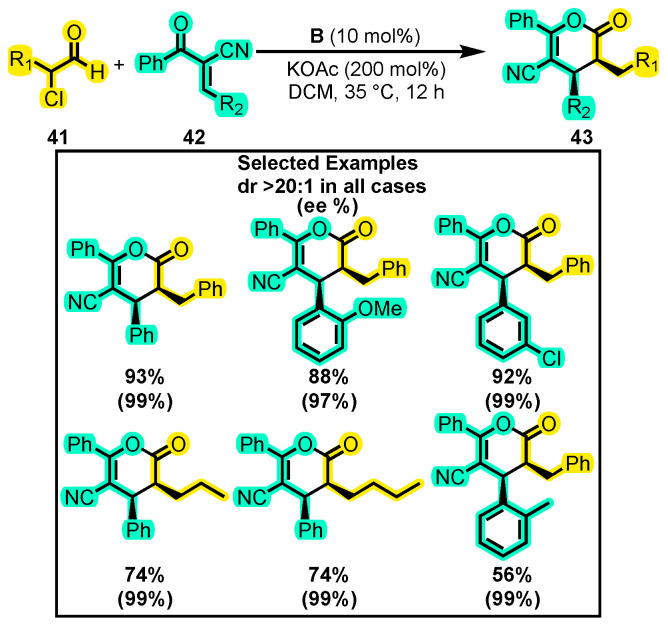
Synthesis of tetra-substituted dihydropyranones from α-substituted aldehydes and α-substituted enones. Adapted from reference [81].

While unsaturated aldehydes are commonly used in NHC-organocatalyzed reactions to produce dihydropyranones, saturated aldehydes with electron-withdrawing groups such as halogens and aryloxy groups have also been explored. In this sense, Li and co-workers [81] synthesized dihydropyranones with four substitutions, including a cyano group for its synthetic versatility [82]. To achieve this, they used an α-chloro-substituted aldehyde **34** with an α-cyano enone **35** as a substrate and **B** as a catalyst (Scheme 20). The optimization of the reaction conditions involved testing various organic and inorganic bases, with KOAc being the most effective, achieving the best yield (93%) and enantiomeric excess (99%). CH_2_Cl_2_ was used as the solvent and catalyst **B** was kept constant throughout the study. Substituent variations on the enone included monosubstituted aromatic rings in different positions, with -Cl, -Me, -OMe, and -F groups, as well as naphthalene and 1,3-benzodioxol, while the aldehyde was mainly substituted with benzylic groups and aliphatic chains containing two to seven carbons. The yields obtained for these substrates were higher than 56% with enantiomeric excesses of up to 99% (dr > 20:1 in all cases). Furthermore, the gram-scale assay showed no significant changes in yield, highlighting the robustness of the reaction.

**Scheme 21:**
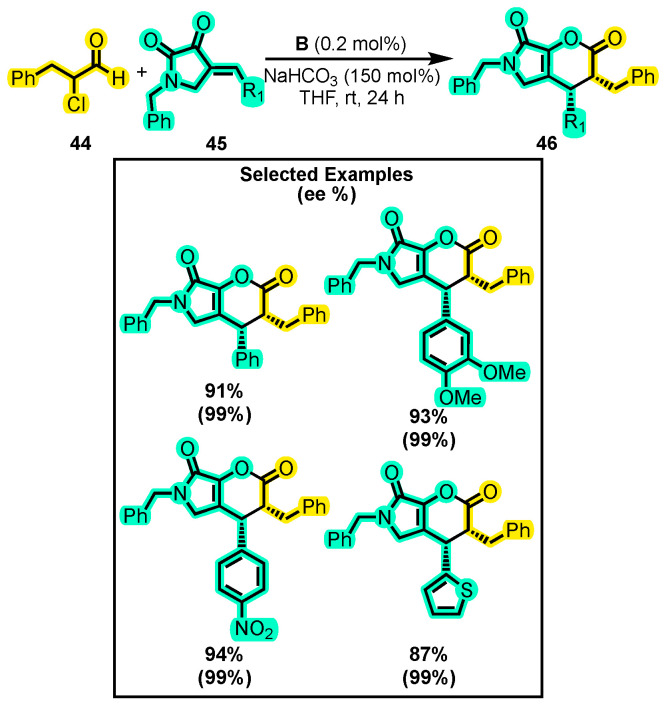
Synthesis of bicyclic dihydropyranones from α-substituted aldehyde and cyclic enones. Adapted from reference [57].

In another study, Li and co-workers reported the synthesis of bicyclic dihydropyranones **46** from α-chloro aldehydes **44** and cyclic enones **45** on a gram scale and with a low catalyst loading (Scheme 21) [57]. They utilized only 0.25 mol% catalyst **B**, which is nine times less than the loading used in many other studies. Additionally, they made no changes to the solvent (THF) or base (NaHCO_3_). The substitutions of an aromatic ring with electron-donating and -withdrawing groups were evaluated in the enone, and the reaction exhibited high enantioselectivity, with an enantiomeric excess greater than 93% for all substitutions tested when **B** was used as a catalyst (Scheme 21). When alkyl aldehydes were used as substrates, good yields were also obtained. The reaction was then scaled up to 1 g of the enone and a catalyst loading of only 0.025 mol%. Despite increasing the reaction time to 48 h, the yield reached 82% with a diastereomeric ratio greater than 99%. This represents a significant advancement in the field of dihydropyranone synthesis as it allows for the production of gram-scale quantities of these compounds with a low catalyst loading (the lowest catalyst loading ever reported in NHC organocatalysis), thus reducing the cost and waste associated with the reaction. The synthesis of bicyclic dihydropyranones involves the fusion of pyrrolidone and dihydropyranone, two important pharmacophore groups that have shown potential for the development of novel drugs [83]. The combination of these groups in a single molecule provides a unique structural motif that can be leveraged to modulate the pharmacological properties of the resulting compounds. This makes bicyclic dihydropyranones an attractive scaffold for drug discovery and development. Moreover, the ability to efficiently synthesize these compounds using NHC catalysis offers a powerful tool for generating structurally diverse libraries of bicyclic dihydropyranones that can be screened for their biological activity.

**Scheme 22:**
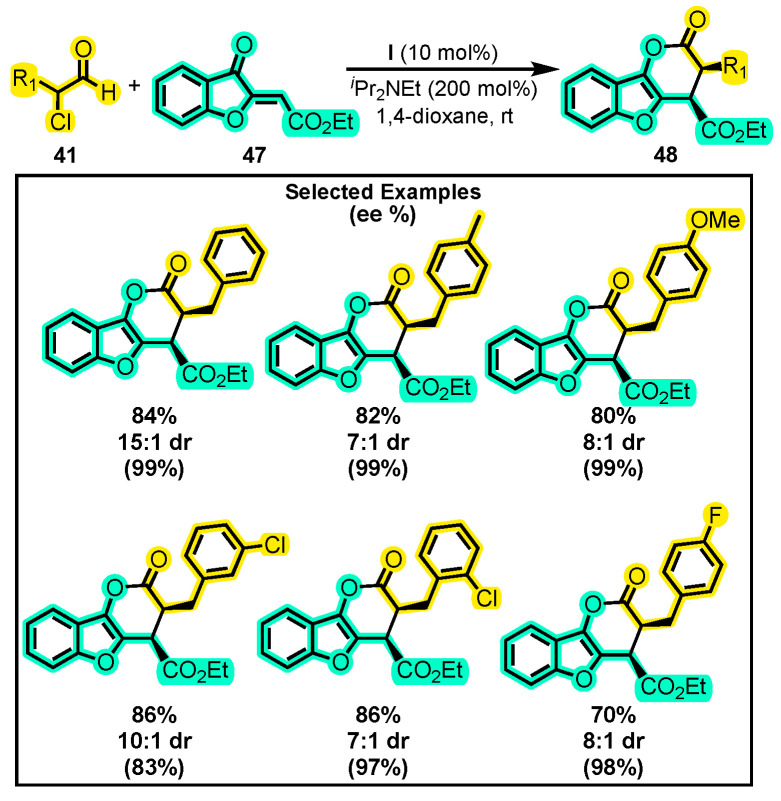
Synthesis of tricyclic dihydropyranones from α-substituted aldehyde and aurones. Adapted from reference [84].

In a related study, a novel [4+2] annulation reaction between α-chloroaldehydes **41** and aurones **47** was developed to form compounds **48** (Scheme 22) [84]. This reaction was performed using *^i^*Pr_2_NEt and 1,4-dioxane as the base and solvent, respectively, since they exhibited the best diastereomeric ratio (dr) and enantiomeric excess in preliminary experiments. The authors observed that the presence of the mesityl moiety in catalyst **I** and its electron-donating ability contributed to an enhanced yield compared to other catalysts. The variations in the substituents of **41** were mainly of two types: (i) *p*-substituted benzyl with halogens, Me, and OMe groups, and (ii) hydrocarbon chains with 2–8 carbons, with the best result being obtained using a hexyl group (95% yield, 7:1 dr, and 98% ee). The high enantiomeric excesses obtained in this study confirmed the high enantioselectivity of catalyst **I**. The coexistence of dihydropyranone with benzofuran presents a promising avenue for the development of molecules with significant biological activity, as benzofuran is present in a large number of natural products known for their bioactivity [85].

**Scheme 23:**
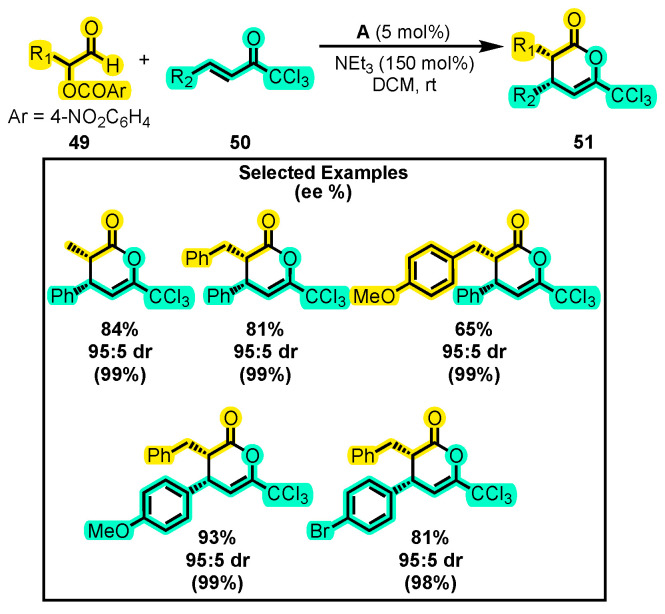
Synthesis of *syn*-dihydropyranones from α-aroyloxialdehydes and trichloromethyl ketone. Adapted from reference [86].

In the search for efficient synthetic methods to obtain *syn*-dihydropyranones, a study was conducted where an α-aroyloxyalkaldehyde **49** was reacted with a trichloromethylketone **50**, leading to the formation of **51** in good yields (Scheme 23) [86]. The reaction was found to be suitable for solvents such as THF, Et_2_O, and toluene, but the best yield was obtained with CH_2_Cl_2_ at a loading of 5 mol% **A**. It was observed that a decrease in catalyst loading leads to a lower product yield. The authors also studied the substrate scope. They found that the reaction took place with different substituents on both **49** and **50** substrates. Nevertheless, the reaction time and catalyst loading required were different in each case. The enantiomeric excess obtained in the study was generally greater than 99%, indicating the high enantioselectivity of the catalytic system studied. Furthermore, one of the isolated products was subjected to an epimerization process with NEt_3_ and CD_2_Cl_2_ at room temperature, which resulted in both *syn* and *anti*-isomers forming in a 67:33 ratio. To further explore the reactivity of the synthesized dihydropyranones, the product was treated with benzylamine and DMAP to induce ring opening, resulting in the formation of diamides, trichloromethyl esters, and diesters with yields of 83%, 50%, and 20%, respectively. The use of trichloromethyl ketones for the synthesis of dihydropyranones has been previously studied due to their ability to undergo processes such as alcoholysis or aminolysis, which facilitate ring opening and lead to the formation of stereodefined diesters or diamides [87].

**Scheme 24:**
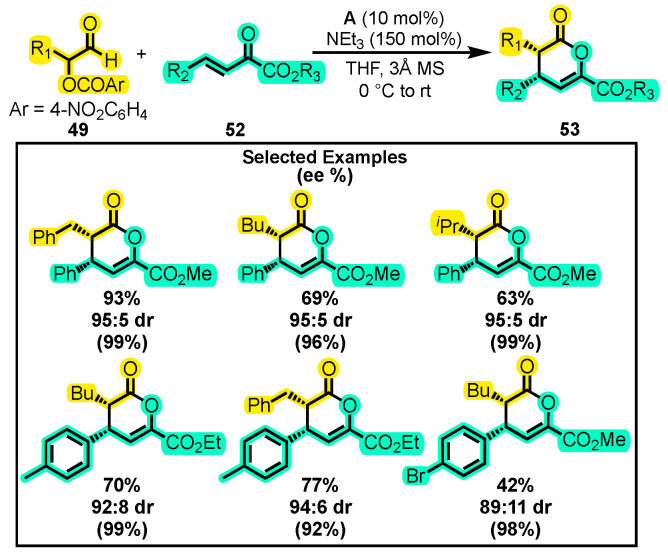
Synthesis of trisubstituted dihydropyranones from α-aroyloxialdehydes and ketoesters. Adapted from reference [88].

On the other hand, Taylor and co-workers developed a novel method for the synthesis of trisubstituted dihydropyranones **53** through the reaction of α-aroyloxyaldehyde **49** and α-ketoester **52** with catalyst **A**, NEt_3_, and THF as a catalytic system (Scheme 24) [88]. Diastereoselectivity was improved at temperatures below 0 °C, but it decreased product yield. Therefore, the reaction was started at 0 °C and slowly warmed to room temperature. The impact of changing the solvent, base, and catalyst was not explored. In contrast, the authors were able to study the substrate scope of the reaction. Thus, substituents including isobutyl, benzyl, and *n*-butyl were evaluated on **49**, while aromatic rings substituted at the *para* position, methyl groups, and ethyl groups were studied on **52**. The authors found yields between 42–93% with reaction times ranging from 3 to 24 h. Subsequently, they performed an epimerization of one product under different conditions using the same base, solvent, and catalyst. Thus, they generated three products with chiral characteristics distinct from those of the initial compound. Another catalytic test achieved yields of up to 90% in only 9 h at room temperature and a catalyst loading of only 5% for the reaction of α-aroyloxyaldehydes and γ-ketoesters. While proline derivatives can catalyze the synthesis of dihydropyranones substituted at position two with esters, an additional hemiacetal oxidation step is necessary [89,90,91,92]. This highlights the superiority of NHC organocatalysis over proline for synthesizing similar molecules.

**Scheme 25:**
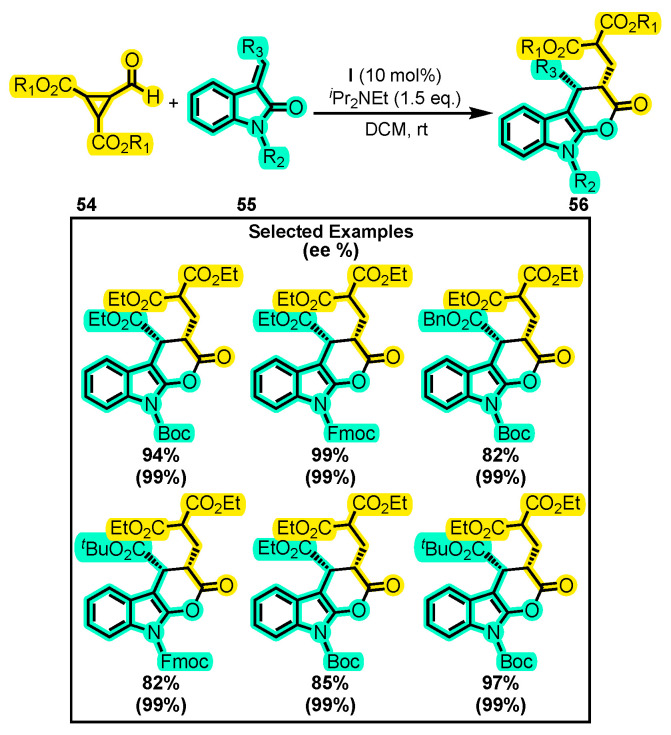
Synthesis of tricyclic dihydropyranones from formylcyclopropanes and alkylideneoxindoles. Adapted from reference [93].

In another report, catalyst **I**, structurally distinct from the previous one, was used in the presence of formylcyclopropane **54** and alkylideneoxindole **55** (Scheme 25) [93]. The resulting tricyclic dihydropyranone **56** was obtained with a yield of up to 99% and an enantiomeric excess of 99%. Various conditions were tested, but the yield was improved by using *^i^*Pr_2_NEt and CH_2_Cl_2_, rather than NEt_3_, K_2_CO_3_, DBU, toluene, THF, and *n*-hexane. As for the substitutions, cyclopropane esters worked well in the reaction with short aliphatic substituents, while substituents in the alkylideneoxindole were mainly carbamates. This demonstrates that the method allows a wide variety of substrates and substituents while maintaining good results, thereby enabling it to be improved and compared with other reported routes for synthesizing compounds with similar structures [94,95].

**Scheme 26:**
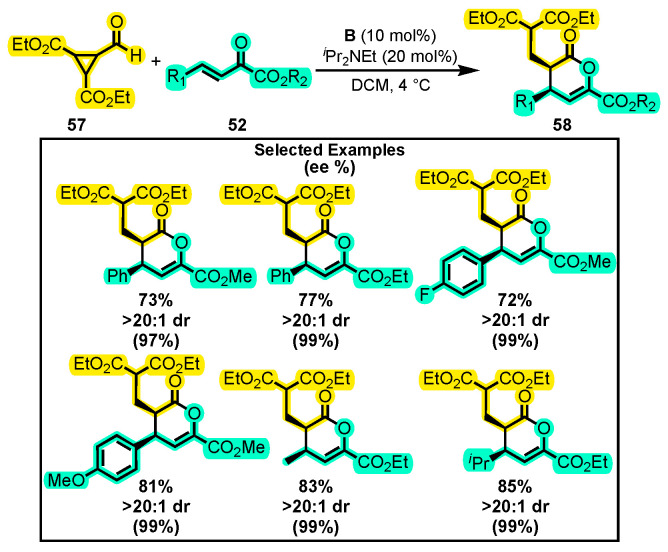
Synthesis of trisubstituted dihydropyranones from formylcyclopropane and α-keto esters. Adapted from reference [93].

The authors also investigated the reactivity of formylcyclopropane **57** towards β,γ-unsaturated α-keto esters **52** containing aryl groups substituted with either electron-donating or electron-withdrawing groups located in different positions to yield the corresponding trisubstituted dihydropyranones **58** using the enantiomer of catalyst **I** (**B**) (Scheme 26). However, the yield decreased in some cases under the same reaction conditions. Notably, they discovered that by using half the amount of catalyst, but increasing the reaction time and ensuring constant stirring, it was possible to achieve comparable results to the full catalyst loading in one of the catalytic runs.

## 4. Ynals and Ketones as Reagents for Dihydropyranones

**Scheme 27:**
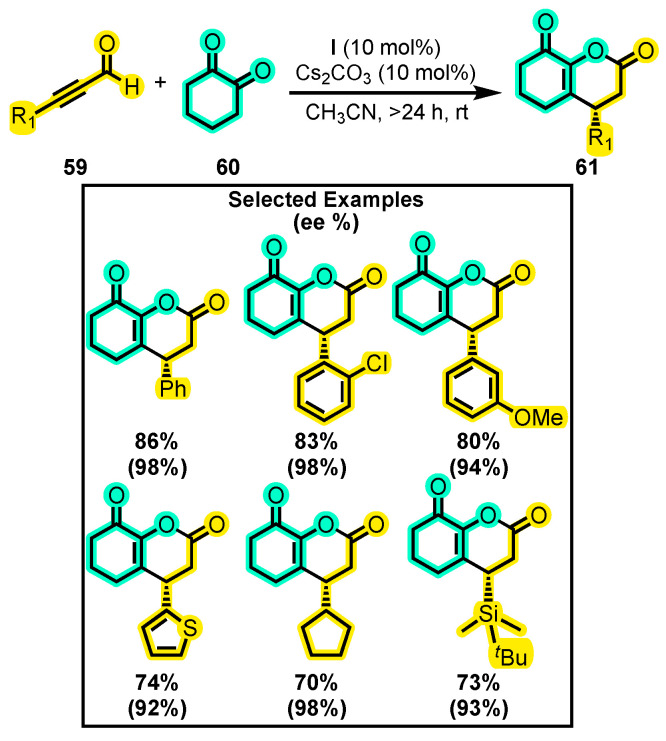
Synthesis of bicyclic dihydropyranones from ynals and a cyclic dicarbonyl compound. Adapted from reference [96].

Although the reactivity of ynals and ketones towards the formation of NHC-organocatalyzed dihydropyranones has not been as extensively explored in recent years compared to that of saturated aldehydes, the introduction of these functional groups adds new structural and electronic features to the molecule’s main nucleus. In this sense, Ren and co-workers investigated these functional groups by reacting an ynal **59** with a cyclic dicarbonyl compound **60** to produce bicyclic dihydropyranones **61** (Scheme 27) [96]. The authors extensively evaluated various reaction conditions, including testing eleven solvents and ten bases. The optimal results were obtained using acetonitrile and Cs_2_CO_3_ at room temperature for 24 h. Although the reactivity was initially tested using **M** as a catalyst substituted with TMS or TBS in one side, and a phenyl group or -C_6_F_5_ moiety in the other side (**M_1_**–**M_3_**), the yield of the final product was less than 14% in all cases, prompting a switch to catalyst **I**. When they tested **I** with BF_4_^−^ as a counterion under the same conditions, a yield of 34% and an enantiomeric excess of 97% were achieved. Conversely, when Cl^−^ was used as a counterion, the yield dropped to 31%, and enantiomeric excess dropped to 81%, demonstrating the significance of the anion in catalytic activity. With the optimized conditions, the importance of the ynal substituent was investigated. The substitutions included a range of diversely substituted aromatic rings, aliphatic chains, and different types of cycles. Longer reaction times of over 24 h were necessary to obtain yields of up to 86% and enantiomeric excesses exceeding 92%. These compounds have attracted the attention of several pharmaceutical industries due to their skeletal structures that bear similarity to that of α-lapachone [97,98], a compound with anti-vascular activity [99]. This discovery could open new avenues for further research into the synthesis of these types of compounds, especially regarding their potential pharmacological activities.

**Scheme 28:**
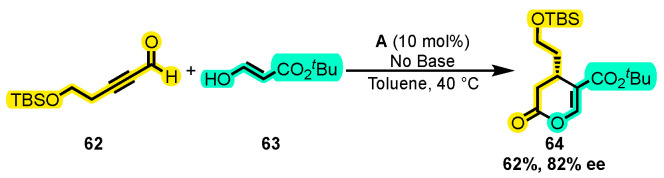
Synthesis of dihydropyran core of oleuropein secoiridoid from a ynal and an α, β-unsaturated ester. Adapted from reference [102].

A novel approach to synthesize secoiridoids derived from oleuropein and elenolide, compounds present in olive oil with important biological activities such as anti-hypertensive, anti-inflammatory, anti-oxidant, and anti-cancer effects [100,101], was developed using ynals by Liu and co-workers. In this study, ynal **62** was reacted with an α, β-unsaturated ester **63** in the presence of catalyst **A** without the need for a base (as the basicity of the Cl^−^ anion was sufficient for the reaction to occur) to produce product **64** (Scheme 28) [102]. This innovative method could lead to the synthesis of a variety of Secoiridoid derivatives with potential biological activities.

**Scheme 29:**
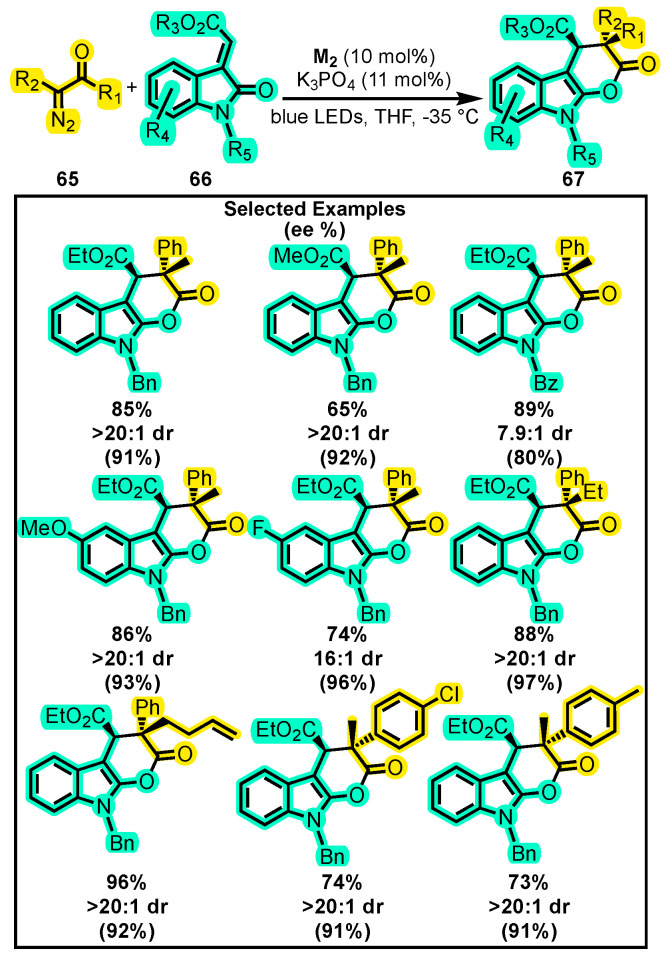
Synthesis of chiral dihydropyranones from α-diazoketones and 3-alkylenyloxyindole. Adapted from reference [104].

Regarding ketone reactivity towards the formation of dihydropyranones, the only report in recent years was published by Wang and co-workers. They developed a unique method for synthesizing dihydropyranones by relying on the Wolff rearrangement [103], which involves the conversion of α-diazoketones to ketenes. The reaction was carried out by reacting an α-diazoketone **65** with a 3-alkylenyloxyindole **66** in the presence of blue LEDs, **M_2_**, K_3_PO_4_, and THF, which served as the activator, catalyst, base, and solvent, respectively, with the aim to improve the yield and enantiomeric excess of products **67** (Scheme 29) [104]. The temperature was also found to be a crucial factor in optimizing the yield and enantiomeric excess. The investigation resulted in the synthesis of over twenty chiral dihydropyranones **67** showing that the reaction had a good tolerance to functional groups and an ample substrate scope. Furthermore, in order to gain insights into the reaction mechanism and intermediates involved in the process, density functional theory calculations have been evaluated [105].

## 5. Carboxylic Acid and Derivatives as Reagents for Dihydropyranones

**Scheme 30:**
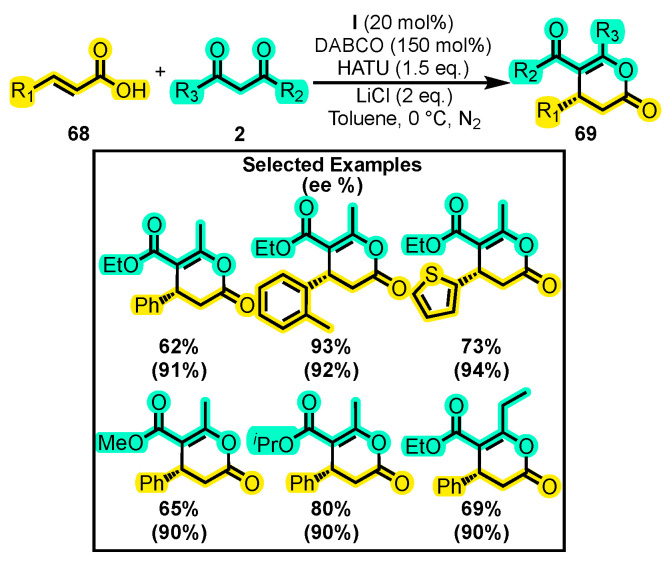
Synthesis of chiral dihydropyranones from α, β-unsaturated carboxylic acid and dicarbonyl compounds. Adapted from reference [106].

In addition to the carbonyl group, NHCs have also been demonstrated to activate carboxylic acids and their derivatives. For instance, Que and co-workers developed a reaction using α, β-unsaturated carboxylic acid **68** and 1,3-dicarbonyl compounds **2** to produce chiral dihydropyranones **69** (Scheme 30) [106]. The reaction employed NHC catalyst **I**, HATU as the carboxylic acid activator, DABCO as a base, LiCl as a Lewis acid, and toluene as a solvent under an N_2_ atmosphere. Notably, aliphatic substitutions at the carboxylic acid did not undergo reaction, whereas substitutions at the 1,3-dicarbonyl compound allowed for various substituents, with ethyl ester derivatives demonstrating the best outcomes. The yields were high, reaching 93%, while the enantiomeric excess was excellent at 94%.

**Scheme 31:**
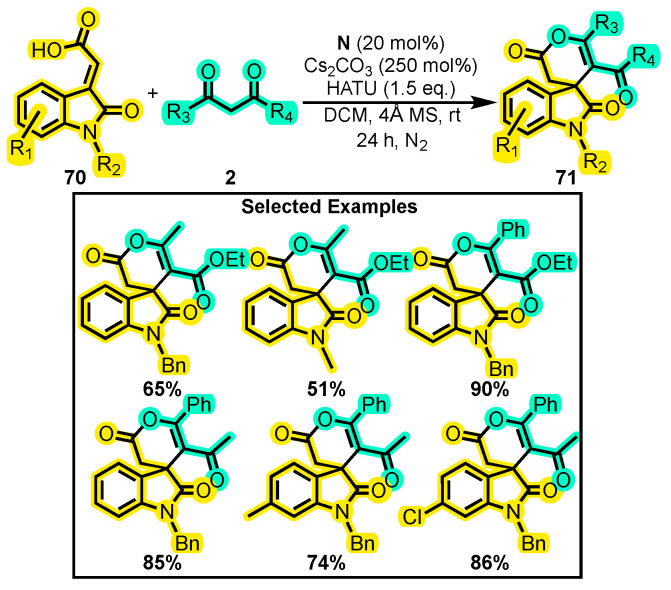
Synthesis of dihydropyranones from isatin-derived carboxylic acids and dicarbonyl compounds by Zhang. Adapted from reference [107].

In a subsequent study, Zhang and co-workers studied the reaction between α, β-unsaturated acid **70** and 1,3-dicarbonyl compounds **2** under an N_2_ atmosphere using Cs_2_CO_3_, HATU, CH_2_Cl_2_, catalyst **N**, and a 4Å molecular sieve. The latter was used to remove water as a driving force of the reaction (Scheme 31) [107]. Thus, they were able to obtain dihydropyranones **71** in moderate to good yields. The authors also explored the scope of the reaction, revealing that the aromatic ring of the carboxylic acid tolerated various halogens at position C5, while *N*-substitutions showed good tolerance towards methyl, allyl, and benzyl groups. Furthermore, the authors demonstrated that good yields of the product were possible if the dicarbonyl substrate was not sterically hindered. Interestingly, the yield of desired product **71** improved when the carboxylic acid was added in excess but decreased when it was scaled up to gram quantities.

**Scheme 32:**
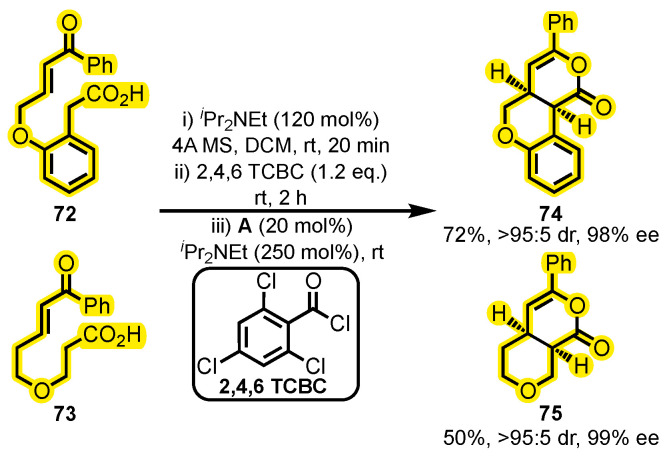
Intramolecular cycloaddition to yield chiral dihydropyranones. Adapted from reference [108].

In contrast to conventional intermolecular reactions, Attaba and Smith recently reported a methodology for the synthesis of two chiral dihydropyranones, **74** and **75,** through an intramolecular cycloaddition of enone and carboxylic acid groups in compounds **72** and **73** (Scheme 32) [108]. The reaction consisted of three steps and utilized catalyst **A**, CH_2_Cl_2_, diisopropylethylamine (*^i^*Pr_2_NEt), and 2,4,6-trichlorobenzyl chloride (2,4,6-TCBC), with a reaction time of 2.5 h. The **74** and **75** dihydropyranones were obtained in moderate to good yields (72 and 50%, respectively) with an excellent diastereomeric ratio and enantiomeric excess. Previous routes for the synthesis of **74** and its derivatives required similar conditions and the presence of a thiourea catalyst [109]. While the yields obtained in these two studies are comparable, the enantiomeric excess achieved with NHCs was higher. On the other hand, analogs of **75** have been the objective of synthetic approaches to the production of calyxin derivatives [110], a group of adducts resulting from the alkylation of diarylheptanoids with a chalcone or flavanone fraction, which have demonstrated broad bioactivity [111].

**Scheme 33:**
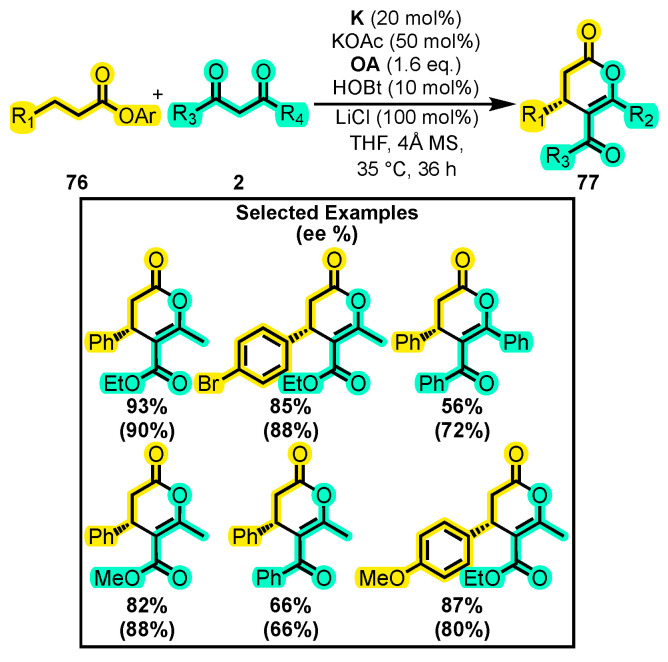
Synthesis of 4,5,6-trisubstituted dihydropyranones from β-substituted esters and dicarbonyl compounds. Adapted from reference [112].

In 2018, a novel study aimed at synthesizing lactams through NHC catalysis was published, but the reaction conditions were later modified to produce 4,5,6-trisubstituted dihydropyranones **77**. The reaction involved a substituted aromatic ester **76** and 1,3-dicarbonyl compounds **2** (Scheme 33) [112]. The authors found that adding hydroxybenzotriazole (HOBt) was crucial to improve the performance and enantiomeric ratio of the protocol. Substitutions with bromide on the aromatic ring maintained the yield, while substitutions on the dicarbonyl substrate using methyl and methoxy moieties showed higher yields than those using phenyl groups. Additionally, the authors found that substitutions with ethyl esters on the 1,3-dicarbonyl compound also produced higher yields. Overall, the modified reaction conditions were effective at producing 4,5,6-trisubstituted dihydropyranones **77** with high yields and enantiomeric excesses.

**Scheme 34:**
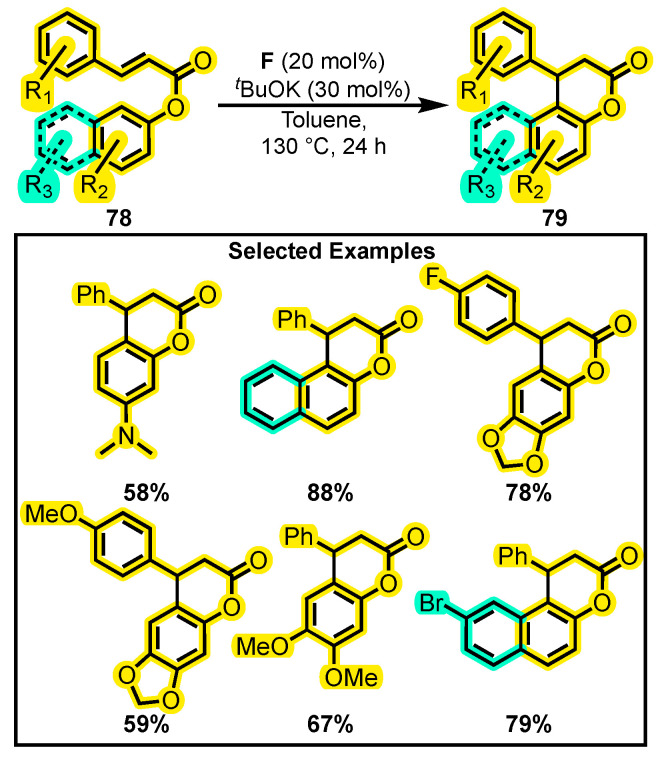
Intramolecular annulation for the synthesis of polycyclic dihydropyranones. Adapted from reference [113].

On the other hand, Zheng and co-workers developed another intramolecular reaction using α, β-unsaturated phenolic esters **78** (Scheme 34) [113]. They evaluated the reactivity of different Lewis bases and acids. The study revealed the significant role of potassium in cooperative catalysis with NHC, leading to good yields regardless of the substitutions. This approach presents a simple, fast, and efficient way of obtaining polycyclic structures with a dihydropyranone core **79**.

**Scheme 35:**
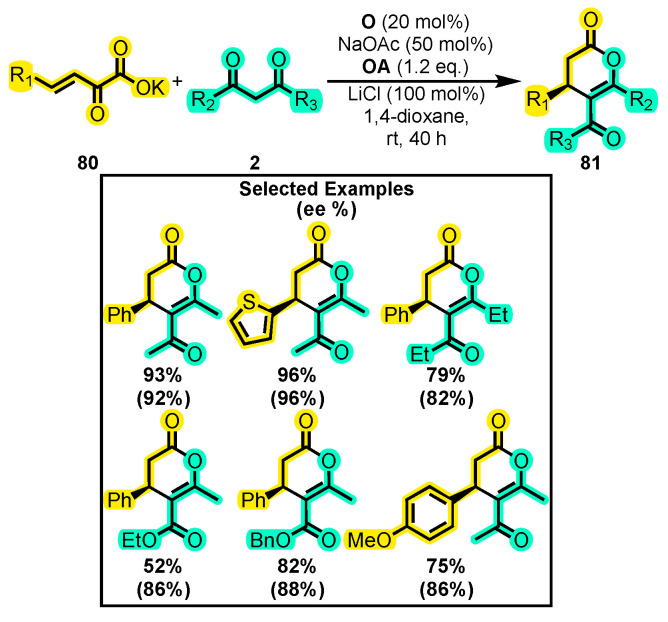
Synthesis of chiral dihydropyranones from potassium 2-oxo-3-enoates and dicarbonyl compounds. Adapted from reference [114].

In another report, Gao and co-workers developed a method to generate various types of products from the homoenolate intermediate, including trisubstituted dihydropyranones, by using the ability of potassium 2-oxo-3-enoates to replace α, β-unsaturated aldehydes in NHC-catalyzed reactions [114]. The reaction involved potassium 2-oxo-3-enoates **80** with 1,3-dicarbonyl compounds **2** to produce chiral dihydropyranones **81** by using the catalyst **O** (Scheme 35). The protocol provided a maximum yield of 96% and an enantiomeric excess of 96% when the substitution at **81** was with a thienyl group and the substitution at the dicarbonyl compound was with methyl groups. However, the yield significantly decreased when using ethyl and esters at **2**.

**Scheme 36:**
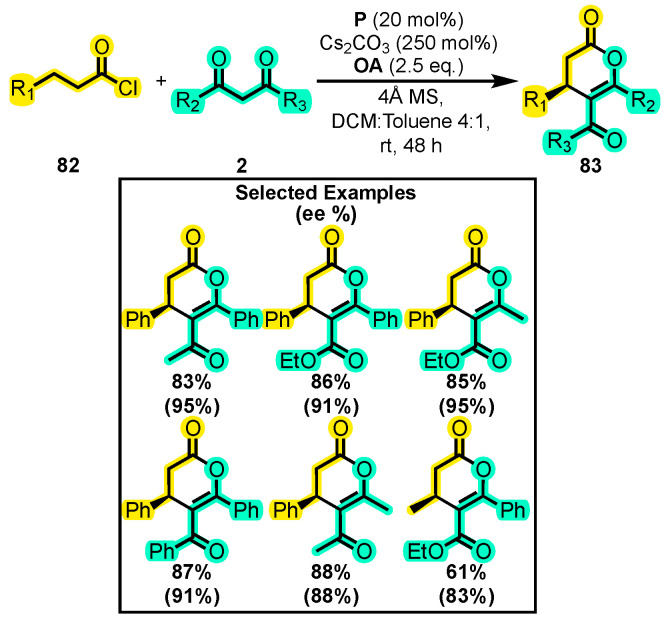
Synthesis of dihydropyranones from acyl chlorides and dicarbonyl compounds. Adapted from reference [115].

In 2019, Zhu and co-workers developed a method to confer electrophilic properties to the β-position of an acyl chloride **82**, enabling its reaction with 1,3-dicarbonyl compounds **2** in the presence of an oxidizing agent, Cs_2_CO_3_, **P** as a catalyst and a mixture of CH_2_Cl_2_ and toluene to produce dihydropyranones **83** (Scheme 36) [115]. The reaction scope was studied with different substituents at the dicarbonyl compound, including methyl, ethoxy, and phenyl groups. Although the yield of the isolated product did not exceed 90%, the enantiomeric excess ranged from 95% to 83%.

**Scheme 37:**
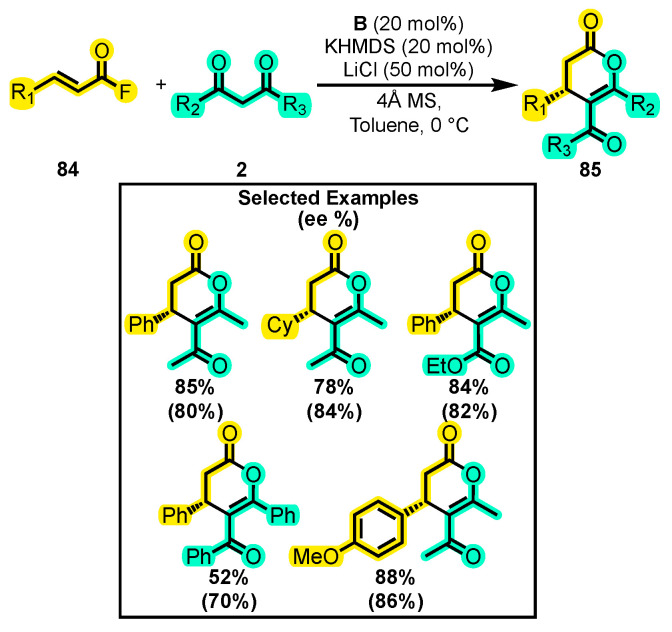
Synthesis of dihydropyranones from acyl fluorides and dicarbonyl compounds. Adapted from reference [116].

In another report focusing on acyl halides, researchers aimed to generate a reactive α, β-unsaturated acylazolium intermediate from acyl fluorides to facilitate various cyclization reactions. In one such reaction, a [3+3] cyclization between an α, β-unsaturated acyl fluoride **84** and 1,3-dicarbonyl compounds **2** was performed using **B** as a catalyst (Scheme 37) [116]. To optimize the reaction conditions, a computational study was conducted to determine the thermodynamic and kinetic feasibility of fluoride trapping by the HMSD generated from the KHMSD (base) and avoid the inactivation of the catalyst by HF. Based on this principle, additions of LiCl and 4Å molecular sieves were made to improve the yield, and subsequently, substitutions on the substrates were tested, resulting in yields up of to 88% of the products **85**.

**Scheme 38:**
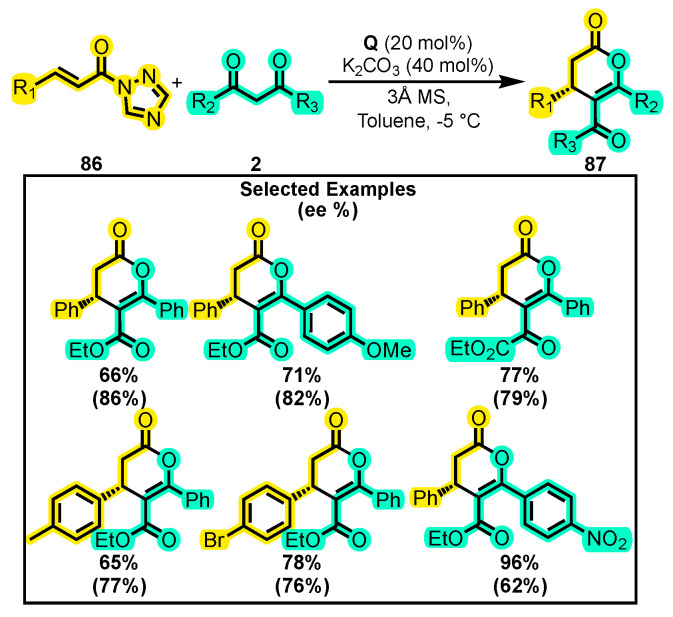
Synthesis of trisubstituted dihydropyranones from *N*-acyltriazoles and dicarbonyl compounds. Adapted from reference [117].

Finally, an alternative method for the generation of the α, β-unsaturated acylazolium intermediate was published by Enders and co-workers. They reported the use of α, β-unsaturated *N*-acyltriazole **86**. This species was able to react with 1,3-dicarbonyl compounds **2** to yield trisubstituted dihydropyranones **87** when **Q** was used as a catalyst (Scheme 38) [117]. However, when the triazole group was replaced by the imidazole or tetrazole group, no reaction or only traces of the product were obtained. In this study, various catalysts, bases, and solvents were explored to optimize the reaction conditions, and the substrate scope was evaluated. The β-position of the acyl triazolium afforded good yields when electron-donating and electron-withdrawing substituents were introduced at the *para* and *ortho* positions of the phenyl ring. However, the enantioselectivity decreased when the dicarbonyl compound was substituted with electron-withdrawing groups.

## 6. Perspectives and Conclusions

The catalytic process using NHC to synthesize 3,4-dihydropyran-2-ones has proven to be exceptionally versatile. The interaction between various functional groups and a wide range of NHCs has enabled the formation of key intermediate species, resulting in the synthesis of simple and fused skeletons and groups. Many of these compounds have the potential for application in biological activity studies due to their relevance in that field. Regarding the chemical structure of the organocatalysts reviewed, it is worth noting that the majority of them were based on triazole rings (**A**–**C** and **G**–**Q**), while only a few were based on imidazole rings (**D**–**F**). The reason for this could be attributed to the electronic properties of triazole-based NHCs, which are generally more nucleophilic than their imidazole-based counterparts [118]. This enhanced nucleophilicity enables triazole-based NHCs to more readily activate α, β-unsaturated and saturated aldehydes, ynals, ketones, carboxylic acids, and derivative compounds compared to imidazole-based NHCs. Thus, it is reasonable to expect that triazole-based NHCs would lead to more active catalytic systems in the formation of dihydropyranones. However, we invite further research to explore the potential of imidazole-based NHCs as organocatalysts in the reactions presented here, as this is a relatively unexplored area. It is also worth highlighting that organocatalysts **A**, **B**, and **K** were the most frequently used in these reactions, possibly due to their commercial availability and, also, the rigidity imposed by their chiral fused rings. This rigidity allows for effective chiral induction from the catalysts to the substrates, resulting in high enantioselectivities (ee > 99%) in several cases. In this sense, the exceptional yields and enantiomeric excesses achieved in various studies underscore the potent catalytic capabilities of NHCs in this type of reaction. Despite the outstanding performance, the process’s development faces certain challenges, such as improving catalyst loading, as only one report has shown loadings lower than 1 mol% [57]. Nevertheless, it has been demonstrated that low catalyst loadings can be achieved with other types of organocatalysts that are different to NHCs [119,120,121,122,123]. On the other hand, the high catalyst loadings observed with NHCs can be attributed to their ability to function not only as effective nucleophiles for activating substrates and initiating catalytic cycles, but also to their potential to activate other substrates present in the reaction medium as Bronsted acids when they are in the form of an NHC precursor (i.e., imidazolium or triazolium salts) [45,124,125]. Therefore, NHCs may play a dual role in some reactions, necessitating the use of high catalyst loadings to ensure optimal activity. On the other hand, the recyclability of the catalyst remains a concern. However, it has been demonstrated that catalytically active species can be observed even after the end of the process [126]. This clearly shows that this species could be used in subsequent processes, especially those of a chiral nature since these are usually expensive. In this sense, an interesting approach that would overcome this drawback would be the heterogenization of these organocatalysts through the tailor-made design and synthesis of, for example, silica- or polymer-supported chiral NHC catalysts.

On the other hand, the formation of NHCs is usually carried out in the presence of a base (at high loadings), so implementing free NHC electrogeneration could be a viable alternative to this problem. While most research has demonstrated a wide scope in their reactions, these molecules are not usually studied for their biological activity. Conducting in silico approximations, along with synthetic development, could improve the probability of success. In addition, based on this review, we propose that easily available and more stable reagents such as carboxylic acids and derivatives, which have been scarcely explored, have significant potential as reagents for NHC organocatalysis. Then, we invite a further exploration of their use. Moreover, determining specific rotation values for the chiral dihydropyranones obtained is highly relevant, as reported by Liu and co-workers [102]. Further research and development may potentially overcome these obstacles and pave the way for the more efficient and sustainable production of 3,4-dihydropyran-2-ones using NHCs as catalysts.

## Data Availability

Not applicable.
